# Knowledge translation of climate change research into public health action: a scoping review

**DOI:** 10.3389/fpubh.2025.1632799

**Published:** 2025-10-27

**Authors:** Chanelle Mulopo, Nyamongo Onkoba, Samuel Abimbola, Ezgi Baltaci, Caradee Y. Wright, Bey-Marrie Schmidt

**Affiliations:** ^1^Centre for Epidemic Response and Innovation, Stellenbosch University, Stellenbosch, South Africa; ^2^School of Public Health, University of the Western Cape, Bellville, South Africa; ^3^Department of Biomedical and Health Sciences, The Technical University of Kenya, Nairobi, Kenya; ^4^Cyprus International Institute of Environmental and Public Health, Cyprus University of Technology, Limassol, Cyprus; ^5^Division Policy and Implementation Research for Cancer Prevention, German Cancer Research Centre (DKFZ), Heidelberg, Germany; ^6^Climate Change and Health Research Programme, Environment and Health Research Unit, South African Medical Research Council, Pretoria, South Africa; ^7^Department of Geography, Geoinformatics and Meteorology, University of Pretoria, Pretoria, South Africa; ^8^Health Systems Research Unit, South African Medical Research Council, Cape Town, South Africa

**Keywords:** climate change and health, knowledge translation, evidence-informed decision-making, public health policy, knowledge to action

## Abstract

**Background:**

There are gaps in translating climate change research into public health action at the global level. Knowledge translation refers to the methods used to bridge the gap between knowledge and action. Given the significant impacts of climate change on health, it is essential to integrate climate research into health decision-making processes. This integration ensures that evidence effectively informs policies and practices at all levels, leading to more timely, equitable, and impactful public health responses.

**Objective:**

This scoping review examines key approaches and identifies gaps in knowledge translation methods for integrating climate change research into public health decision-making.

**Methods:**

A systematic search of the literature was conducted in PubMed, CINAHL, and Scopus using a predefined search string to identify studies on climate change and health published between January 1, 2003, and March 1, 2024, with no geographic restrictions. Retrieved articles were screened and data extracted using Covidence software. Thematic analysis was performed in ATLAS.TI employing Braun and Clark’s six-step method. The identified knowledge translation approaches were categorized according to the Cochrane Knowledge Translation Framework.

**Findings:**

Our findings highlight five primary knowledge translation approaches: (1) monitoring the coverage of climate change and health across media, scientific literature, and government responses; (2) engaging citizens of all ages in participatory activities to address local climate challenges and co-develop policy solutions; (3) integrating knowledge generation, synthesis, and dissemination for effective communication; (4) emphasizing advocacy and education to foster collaborations and gain support from decision-makers; and (5) leveraging health impact assessment tools to guide decision-making related to climate change and health. Nonetheless, we did not find any primary studies on climate change research and knowledge translation in Low- and Middle-Income Countries (LMICs).

**Conclusion:**

There is a need for primary studies on the knowledge translation of climate change research, especially in relation to adaptation, into meaningful public health actions that can inform decision-making and contribute to building climate-resilient health systems in LMICs.

## Background

Climate change poses significant and growing threats to human health, affecting disease patterns, food security, air quality, and the social and environmental determinants of health (1–5, 49). Given the profound impacts, there is an urgent need to ensure that climate change research is effectively integrated into public health decision-making processes (6, 7, 48).

While the terms knowledge translation and knowledge mobilization are often used interchangeably, they represent conceptually distinct approaches to bridging the gap between research and action. Knowledge translation refers to the process of synthesizing, contextualizing, and applying research evidence to improve policies and practices (8, 52). It typically involves targeted efforts to make scientific findings accessible, relevant, and actionable for decision-makers. In contrast, knowledge mobilization encompasses a broader, more participatory process. It emphasizes engagement, collaboration, and the co-production of knowledge with a wide range of actors, including policymakers, practitioners, researchers, and communities-aiming to drive context-sensitive, equitable, and sustainable change (9, 10).

To date, the health impacts of climate change have often been addressed primarily through an environmental lens (50, 51, 53), which has led to a narrow framing of the issue and overlooked its broader implications for planetary health (11–13). Planetary health is an interdisciplinary field that explores the interdependence between human health and the health of natural systems, focusing on how human-induced environmental disruptions affect both (14). Climate change, driven largely by anthropogenic activities, is one of the nine planetary boundaries identified by scientists as thresholds that should not be crossed to avoid irreversible environmental and health consequences (15).

Bridging the gap between climate change research and public health action thus requires more than just the translation of evidence into policy. It also necessitates mobilizing knowledge through inclusive, cross-sectoral, and community-engaged processes. This dual approach-knowledge translation and knowledge mobilization, is critical for developing health responses that are not only scientifically sound but also socially accepted and locally relevant (54).

Unfortunately, this integration remains limited, particularly in low-and middle-income countries (LMICs), where vulnerable populations, least responsible for climate change-bear a disproportionate burden of its health impacts (16). These populations are often excluded from the design of climate and health policies, resulting in responses that fail to address equity and contextual realities (17, 18). While knowledge mobilization is critical and would be beneficial in LMICs, exploring both concepts is beyond the scope of this manuscript; hence, the focus of this manuscript is on knowledge translation, which focuses on how climate change and health research inform decision-making.

The 2023 UNEP Adaptation Gap Report underscores this issue, highlighting that the incorporation of knowledge translation into public health decision-making is still insufficient at the global level (19). Yet, knowledge translation is critical for strengthening health systems’ resilience to climate-related health threats-both current and emerging (20).

To be effective, knowledge integration must follow a transdisciplinary knowledge mobilization approach, fostering collaboration among health professionals, environmental scientists, policy-makers, educators, and affected communities (20, 21, 55). In addition, embedding climate-health content into public health education and training programs is essential. This not only promotes the exchange of knowledge but also supports two-way, reciprocal learning that recognizes local expertise and promotes the co-creation of solutions.

Despite growing awareness of the need for integrated action, there is a lack of comprehensive evidence on how climate change research is being translated into public health strategies and interventions.

## Aim of the review

This coping review aims to:

(a) Provide a synthesis of research on the knowledge translation of climate change and health evidence into public health action;

## Methodology

A previously published scoping review protocol (22), grounded on Arksey and O’Malley’s methodology (23) and further refined using the Joanna Briggs Institute (JBI) framework, was followed. The review adhered to nine systematic steps: (1) defining the research question; (2) establishing inclusion and exclusion criteria; (3) delineating the search strategy; (4) executing the search; (5) selecting evidence; (6) extracting data; (7) charting the evidence; (8) summarizing and reporting the findings; and (9) consulting stakeholders (24). Findings are reported following the PRISMA Extension for Scoping Reviews (PRISMA-ScR) (25).

### Defining the research question

The research question for this review was refined through collaborative team consultations to investigate the relationship between knowledge translation, climate change, and health from a planetary health perspective. Knowledge translation was operationalized within the framework of knowledge into action, practice, or informed decision-making (26). The specific objectives were to (a) explore the relationship between knowledge translation, climate change research, and health decision-making, (b) identify climate health risk focus areas presented in health decision-making, and (c) lastly, map the knowledge translation strategies that are being implemented to advance evidence-informed decision-making in climate-health research.

### Inclusion and exclusion criteria

The PCC (Population, Context, Content) framework (27) guided the inclusion criteria, emphasizing three key groups: (1) evidence producers including researchers from universities, research centers, climate/environmental networks, or governments; (2) evidence users, such as healthcare providers, public health specialists, environmental and health practitioners, policymakers, climate change activists, non-governmental organizations, Community-Based Organizations (CBO), and civil society movements; and (3) intermediary organizations, such as knowledge brokers and implementation research institutions, globally. The review’s core concepts centered on climate change as an intervention and health decision-making as an outcome. Eligible studies were required to address climate change, health, and knowledge translation or health decision-making, with no geographical restrictions and a publication period spanning 2003–2024.

### Searching for evidence

To identify relevant peer-reviewed and gray literature on knowledge translation, climate change, and health decision-making, a comprehensive search strategy was developed in collaboration with a public health subject librarian at the University of the Western Cape (UWC). The strategy was applied across PubMed, CINHAL, and Scopus and supplemented by gray literature from repositories such as the World Health Organization (WHO), the Planetary Health Alliance, and the University of the Western Cape electronic dissertations and theses repository. The exclusion of other databases such as Web of Science or Embase was based on consultations with the Subject Librarian. Based on the searches, PubMed, CINHAL, and Scopus emerged as the top databases where we would find the articles that met our search criteria. Search strings (detailed in Supplementary Box 1) were customized for each database to ensure thorough coverage of relevant studies. Given the increasing interest in the impact of climate change on health over the past two decades, the search was restricted to studies published between 1st January 2003 and 1st March 2024 based on the assumption that there has been an increase in the number of publications on climate change and health in the last two decades. Studies were excluded if they failed to address all three main concepts of interest (i.e., climate change, health, and knowledge translation), did not demonstrate how climate-health research was translated into policy and action, or was not published in English.

### Selecting the evidence

Article screening was conducted using Covidence^1^ in two phases: title/abstract and full-text review. Both phases were performed independently and in duplicate by review authors (CM, SA, and NO) to ensure the accurate identification of potentially eligible studies.

### Extracting the evidence

Data extraction was performed independently and in duplicate using Covidence, with two reviewers assigned per article. The standard Covidence template was adapted to align with the study’s objectives. Four reviewers (CM, SA, NO, and EB) contributed to the data extraction process, with EB recruited and trained to replace BS for this task. We first piloted the data extraction template by extracting data from the same article independently; we then met over Zoom to compare our templates and discussed inconsistencies, clarified issues, and adjusted the template for the actual data extraction. During the data extraction process, the author, CM, continuously checked the data extraction process in real time on Covidence and would engage with the authors where necessary. After data extraction was complete, CM compared the templates of two independent reviewers side by side and checked for inconsistencies or missing data. Once the quality check was complete, the extracts were copied into ATLAS.TI.^2^ Extracted data included study characteristics (author, study aim, study design, geographical location), knowledge translation approaches utilized, climate-health risk focus areas, the stakeholders engaged in the study, and key study findings.

### Coding analysis and synthesis

The authors (CM & NO) reviewed the extracted data and developed a coding frame informed by the research objectives. Data analysis took place in ATLAS.TI, using Braun and Clark’s six-step thematic analysis methodology: (i) familiarization with data, (ii) generating initial codes, (iii) searching for themes, (iv) reviewing themes, (v) defining and naming themes, and (vi) writing up the results (28). To present the identified knowledge translation approaches, the Cochrane Knowledge Translation Framework was employed. We applied the Cochrane Knowledge Translation framework to classify the different knowledge translation activities presented in the literature on climate change and health. The Cochrane Knowledge Translation framework outlines six themes, which are:

1. Prioritizing and co-production of evidence: This theme focuses on the creation of evidence that addresses the specific needs of the users, ensuring that the research produced is relevant and meets the priorities of those who will use it.
2. Packaging, push, and support to implementation: This refers to ensuring that the users receive and can act on the evidence and products.
3. Facilitating pull: Refers to growing the user’s capacity to find and use the evidence.
4. Exchange: Engaging with the users to support their evidence-informed decision-making.
5. Improving climate: Advocating for evidence-informed decision-making.
6. Sustainable knowledge translation process: Building a sustainable infrastructure for knowledge translation.

Therefore, for each approach identified in the literature, we looked at how many of the Cochrane knowledge translation activities were met.

## Results

**Figure 1:**
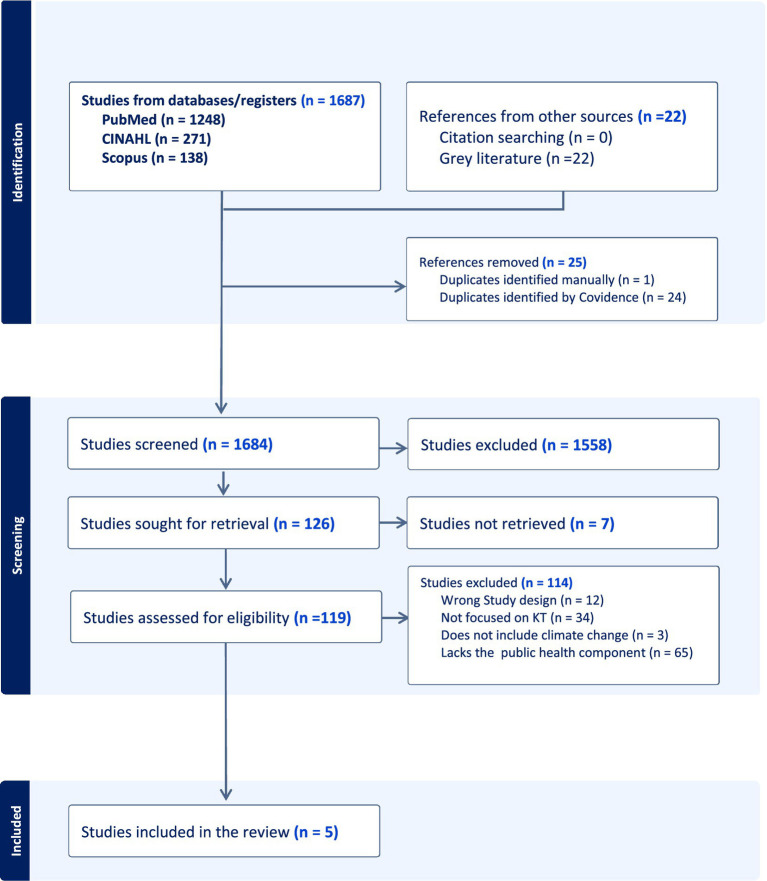
PRISMA flow diagram illustrating the selection process of included studies.

Five studies met the inclusion criteria and were included in the review, each exploring the three concepts of interest: knowledge translation, climate change, and health (Figure 1). In line with the review objective, we examined key knowledge translation strategies reported in the included studies including engagement activities shaping climate health decision-making.

The studies represented diverse geographic contexts: two were conducted in Australia, one in France, one in Canada, and one was a multi-country study spanning the Netherlands, Portugal, the United Kingdom, Slovenia, Italy, and Poland. Methodological approaches varied, with two employing qualitative methods (29, 30), one using a quantitative design (31), and two adopting mixed-methods designs (32, 33). Although the search strategy covered publications from 2003 to 2024, all included studies were published between 2020 and 2024, reflecting a recent focus on translating climate change and health research into policy and practice (Table 1).

**Table 1:** Summary of included studies that met the criteria of climate health and decision-making.

Authors, date	Country	Aim of the study	Study design	Study population	Climate change exposure and health outcomes	Knowledge translation approach	Study outcomes	Key findings
Beggs et al. (31)	Australia	To track progress on the links between public health and climate change across five key domains using updated indicators and refined analyses in the fifth year of the MJA–Lancet Countdown collaboration.	Quantitative	Indigenous Communities in Australia	Environmental exposures and climate-related extreme events, such as repeated or prolonged exposures to heat and heatwaves, bushfires and smoke, droughts and floods, and changing risk of infectious diseases.	Tracking of national media coverage on climate change and health Tracking of scientific publications on climate change and health Tracking government engagement and involvement in Climate Change and Health Decision-making	Health outcomes (i) Heat impacts on physical and sporting activities (ii) Bushfire adaptations (iii) Indirect impacts such as mosquito-borne diseases and population displacement due to weather-related disaster	There is a need for further decarbonization and clearer policy goals at the federal level to support the transition to clean energy, sustainable transportation, clearer frameworks, and a more sustainable health sector.
Dam et al. (32)	Victoria Australia	To explore how local governments in Victoria, Australia used research to integrate climate and health in public health planning, and the role of collaboration, using survey and interview data.	Mixed-Methods	Local Government Authorities	Integrate a Climate lens into Health planning	Invest time and resources in advocacy and educational work Create Legislation Tailoring communication to the needs of different stakeholders Use local data to build your case Decision-makers should have an appetite for research Form of collaborations and partnerships to foster stakeholder buy-in;	Lack of optimum use of research in decision-making due to (i) time and resource constraints, (ii) climate change being a contested topic, hence requiring advocacy and education to facilitate buy-in, and (iii) the role of research appearing to be secondary to other forms of evidence Collaboration was more associated with research access than research use.	The importance of sharing research in various ways to meet the diverse needs of local government stakeholders. Legislation, while important, was less sufficient in shifting beliefs Emphasis on cross-council engagement in regions that share common climate risks to develop common strategies to address them
Deloly et al. (29)	Rennes France	To support urban transformation for sustainability and health using quantitative health impact assessment, based on Complex Urban Systems for Sustainability and Health project.	Qualitative (case study)	Community	Support the transformation of cities to meet environmental imperatives and to improve the health and wellbeing of current and future population	Health impact assessment (HIA) tool (The results from the tool were used to inform decision-making processes)	Influence policy Build partnerships	The interactions between researchers and stakeholders resulted in building trust between the stakeholders.
El Amiri et al. (30)	Canada	To reflect on the activities of the Working Group on Climate Change and Health, and examine its evolution from a working group into a global community of practice focused on enhancing knowledge brokering in climate change and health across inter- and transdisciplinary fields.	Qualitative	Emerging scholars, researchers, and practitioners	Physical and mental health	Establish a community of practice (knowledge generation, synthesis, and mobilization) Knowledge brokering Community of practice/a platform for knowledge exchange	Establishing a community of practice The working group was established to be an integrated platform of webinars, young professional training and networking institute, professional knowledge mobilization, and dialogue workshops, bibliographic development, and topic-specific briefing notes as a foundation for advancing climate change and health knowledge creation, dissemination, and collaboration	The greatest challenge to climate action in public health is the lack of opportunities for collaborative engagement and mutual learning between health researchers, practitioners, and other disciplines and sectors The working group members were volunteers, so there was a need for flexibility and realistic timelines because of other competing interests.
Fogg-Rogers et al. (33)	Amsterdam-Netherlands, Aveiro-Portugal, Bristol-U.K., Ljubljana-Slovenia, Liguria-Italy, Sosnowiec-Poland	To evaluate public engagement strategies used in the ClairCity project to involve diverse citizen groups in climate and air pollution policymaking, assess changes in understanding and behavior, and explore bottom-up approaches for rapid policy transitions.	Mixed-Methods	Citizens of all ages	Air pollution	Six engagement methods were used: Skylines Game Mobile applications School Competition My city videos Citizen Delphi process Mutual Learning (MLW), Stakeholder Dialogue, and Policy Workshop	Enjoyment Knowledge Behavior	Improving public engagement around CC is essential to raise awareness of health and social impacts and for co-designing fair policy solutions.

### The relationship between climate change research translation and health decision-making

We have identified five main approaches to knowledge translation in the included studies. These approaches offer insights into how climate change and health research is being translated into practice using various engagement strategies. These approaches were: (i) engagement with citizens (33); (ii) establishing a community of practice (30); (iii) the incorporation of climate change into public health planning (32); (iv) using health impact assessment tools to inform decision-making and (29); (v) tracking climate change and health outputs vis-a-vis engagement by decision makers (31).

The multi-country study by Fogg-Rogers et al. (33) provided a strong example of citizen engagement and public communication in shaping local climate and health responses. This study used various participatory tools, such as games, mobile applications, school competitions, videos, mutual learning, stakeholders’ dialogue, and policy workshops, to foster bottom-up approaches for co-developing rapid policy transitions in response to climate change. The authors highlight the importance of designing public engagement activities that are enjoyable and tailored for different audiences. Participants reported that the more they enjoyed the activities, the more they understood the need for emission reduction. Consequently, enjoyable activities resulted in increased knowledge about air pollution, which led them to pledge to change their behavior as individuals or as a community to reduce carbon emissions.

In another study, Dam et al. (32) explored the relationship between climate change and health decision-making, specifically how research is used to inform public health planning and strategy development by focusing on the experiences of local government authorities as decision-makers and using local data and advocacy as a strategy to incorporate climate change into health planning. Their findings highlighted the importance of integrating climate research into the decision-making process to ensure that public health systems are prepared to address the challenges posed by climate change. They used methods such as collaboration, partnership, and advocacy to foster buy-in. This study focused on access to and utilization of research findings to inform policy and public health strategies. The authors reported several challenges during this process, such as the lack of optimal use of existing guidelines in decision-making. While the actors (local government authorities) acknowledged the importance of using research to inform their decisions, they were time and resource-constrained to engage with literature and tackle distinct priorities. Other factors reported to influence the application of research involved contextual factors such as attitudes of decision-makers toward science, legislative requirements, and evidence preferences of decision-makers and practitioners. Furthermore, climate change is a contested topic in Australia, hence requiring advocacy work and education to inform understanding and foster buy-in. It was further reported that at times, the role of research appeared secondary to other forms of evidence. Lastly, although collaboration was reported to be important, it was more associated with evidence access and not evidence use.

The role of communities of practice as a knowledge exchange platform for researchers and decision makers was emphasized in the study by El Amiri et al. (30). This approach began with the organic development of a working group, which later evolved into a formal community of practice. The aim was to elevate knowledge brokering and expand it globally, integrating multidisciplinary perspectives to address climate change and health issues. The working group was established to implement a coordinated and integrated platform of webinars, young professional training and networking institutes, professional knowledge mobilization and dialogue workshops, bibliographic development, and topic-specific briefing notes as a foundation for advancing climate change and health knowledge creation, dissemination, and collaboration. However, since the working group members were volunteers, flexibility and realistic timelines are essential for the working group’s success since its members have competing priorities.

The use of a risk assessment tool to inform policy was another approach reported in the literature. Deloly et al. (29) examined the connection between climate change and health through the lens of conducting policy-relevant research that supports the transformation of cities to ensure environmental sustainability and improve the health of both current and future populations. This approach advocates for using assessment and support tools, illustrated by a case study, to guide decision-making. While the tool did not directly inform policy as intended, it facilitated early collaboration among researchers, decision-makers, and other stakeholders, which was seen as foundational for future influence.

Lastly, Beggs et al. (31) described how tracking climate-health coverage across media, literature, and government activity can shape public and policy awareness. No direct communication with citizens or decision-makers occurred. This approach highlights the importance of tracking and reporting on the progress of climate change and health integration into decision-making to ensure that public health policies align with evolving climate realities. However, the challenges experienced in Australia at the time were the absence of a national health and climate change adaptation plan, which hindered Australia’s preparedness for the impacts of climate change, putting the health and lives of Australians at risk. Furthermore, another challenge is the difficulty in quantifying the impact of climate change on Australia’s indigenous population.

### What are the climate health risk focus areas mentioned in climate health decision-making?

All five studies identified the various health impacts of climate change, addressing a wide range of topics. These include the effects of exposure to extreme weather events, such as heatwaves and floods (31), the impact of climate change on both physical and mental health, (30), the role of air pollution, (33), integration of a climate lens into health planning to address climate change’s adverse effects on communities, including heat stress, floods, sea level rise and climate anxiety (32), and the transformation of cities to mitigate climate change’s effects and improve population health and wellbeing (29). Extreme weather events such as heat waves were found to be associated with high rates of hospital admissions, increased demand for ambulance services, and mortality in Australia (31, 32). Repeated flooding, which caused significant harm to physical and mental health, also contributed to heightened mortality rates in affected regions (31, 32).

Bushfires were reported to have profound impacts on population health, leading to mental trauma due to proximity to the fires or loss of homes or businesses, as well as forced displacements (31, 32). The loss of ecosystems, another consequence of climate change, has contributed to the spread, emergence, and outbreaks of communicable diseases such as the mosquito-borne Japanese Encephalitis Virus (JEV) (30), which further burdens the healthcare systems. Air pollution, particularly from carbon emissions, was identified as a major public health risk. One of the studies suggested that citizen-led initiatives to reduce carbon emissions could be effective if citizens were actively engaged in leisure and recreational activities, raising awareness of air pollution and its impacts on both the environment and health (33).

### Knowledge translation approaches implemented to promote the uptake and use of climate change research in health decision-making

**Figure 2:**
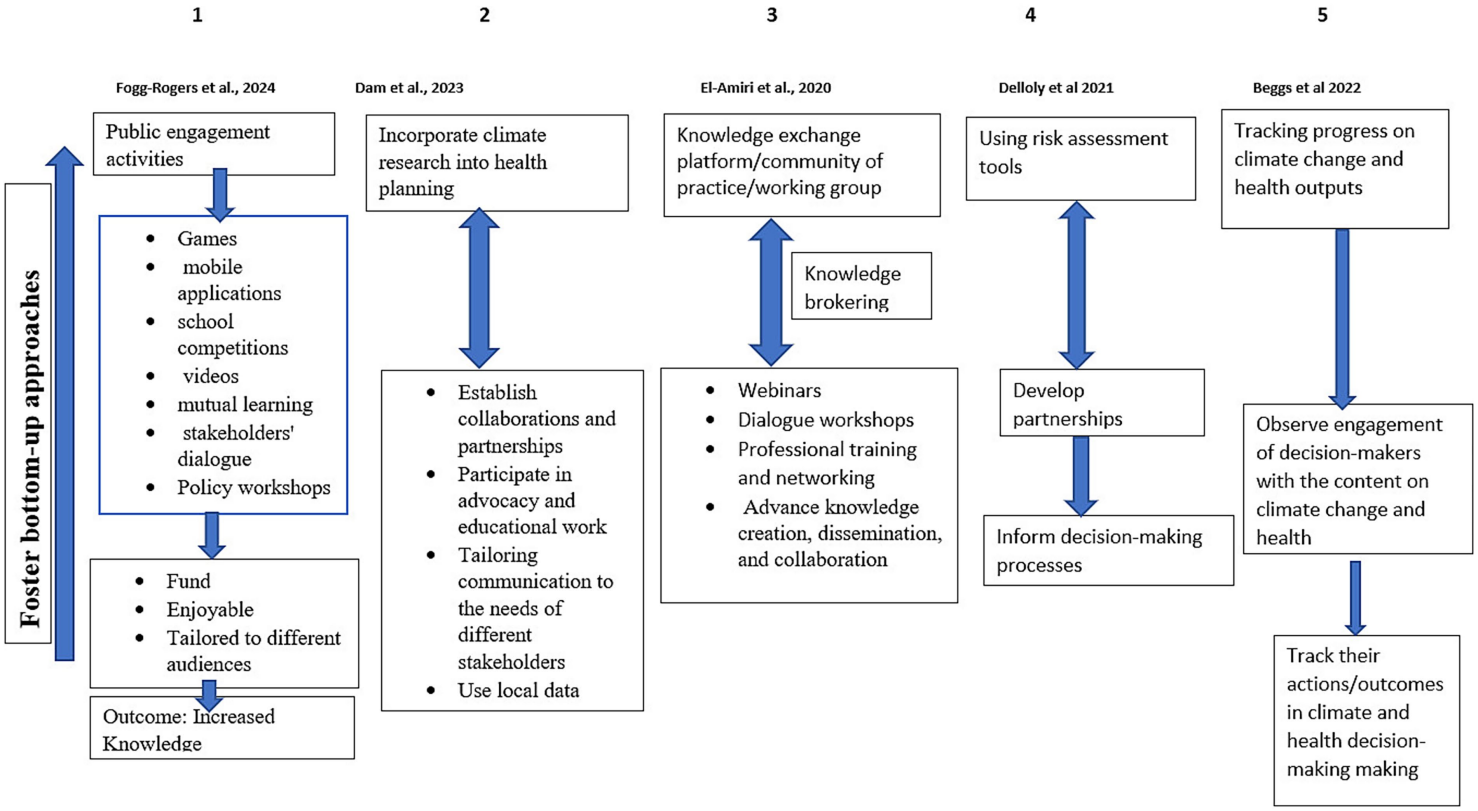
A summary of the knowledge translation approaches identified in the literature.

We categorized the knowledge translation approaches identified in the five included studies based on the Cochrane Knowledge Translation framework (Figure 2). Dam et al. (32) highlighted six facilitators of climate evidence use in decision-making. Regarding prioritizing and co-production, the authors highlighted the importance of *building localized knowledge* on the different climate issues affecting the communities and how to address them. This was achieved through consultations and engagements with decision-makers and key opinion leaders in the community, such as councilors and community elders, as opposed to using research to make a case for a particular action.

Packaging, push, and support to implementation: Tailoring communication to the needs of different stakeholders to get decision-makers’ attention was reported to be common practice. Outputs from community consultations and local data were more influential for decision-makers. Facilitating pull: Although decision-makers were more likely to be convinced to act when there was local data available, there was a direct influence of research in decision-making when the appetite for research was high among decision-makers. However, decision-makers reported a “gap” between the knowledge they gathered and “being able to use it in decision-making processes. Exchange: The authors reported using different approaches of engagement such as networks, partnerships, alliances, workshops, forums, and working groups and also highlighting that there is no one-size-fits-all approach. Different forms of collaboration were crucial in fostering stakeholder buy-in and promoting strategic and coordinated action in public health planning in the context of addressing climate and health. Additionally, internal collaborations with peers and professional networks were identified as drivers of facilitating awareness of and access to research on climate change. Improving climate: By investing time and resources in advocacy and educational work- decision-makers are more likely to engage in well-accepted issues because climate change is a contested topic among people, thus, ample time and resources should be allocated for advocacy and education to facilitate buy-in from decision-makers. Sustainable knowledge translation process: Legislation plays a prevailing influence on decision-making; where a legislative framework on climate change exists, it adds weight to persuading decision-makers to do something about it as it is a requirement in the legislation.

The second knowledge translation approach identified in the literature was the study conducted in Canada (30). This study met four key themes of the Cochrane Knowledge Translation framework. A community of practice was established as a knowledge exchange platform. Regarding prioritization and co-production: this was achieved through the community of practice where members were encouraged to collaborate, exchange, and generate knowledge, the community of practices was responsible for knowledge brokering. Packaging, push, and support to implementation: knowledge synthesis and mobilization was also another important activity. This is evident in the development of short summaries on the impact of climate change on health, briefing notes on the current state of gaps in knowledge associated with climate change and infectious diseases, etc., and a training module that was piloted in Tanzanian Universities and other organizations. Exchange: Webinars were identified as a tool that provided space for a two-way knowledge exchange platform between researchers and practitioners. In this study, the webinars were used to share expert knowledge, recorded and published online alongside other resources on climate change such as summaries on the direct and indirect impacts of climate change and health, serving as a repository for interested parties. The formulation of global partnerships and working groups for knowledge exchange and capacity-building activities such as workshops, panel discussions, and open sessions were regarded as ways of translating knowledge. Collaboration among partners and interested parties was identified as being pertinent. Collaboration could be either lateral or vertical where lateral collaboration refers to reaching out to other academics and professional colleagues locally and internationally whereas vertical collaboration refers to consultations on the strategic global health research priorities for a country in this case Canada.

Sustainable knowledge translation process: The development of the community of practice and enhanced collaboration resulted in knowledge sharing and enhanced capacity through coalition institutes. Other benefits include facilitating access to resources, mentorship, training, collective learning, and opportunities to take part in new initiatives. However, limited emphasis was attributed to equity and inclusion: equity in the efforts to enable and support different worldviews, perspectives, and ideas as an active part of climate health discourse, such as multi-species and indigenous ways of knowing, and inclusion in our efforts to actively bring together practitioners and academic researchers as well as engage with other disciplines and partners from the Global South and Indigenous communities.

The third knowledge translation approach was identified in the study by Beggs et al. (31) which involved tracking coverage of climate change and health topics on various platforms such as (i) social and mainstream media coverage (ii) scientific publications, (iii) government engagement in decision-making on the topic, and (iv) availability of climate change funding for climate change and health research. This approach only met two themes of the Cochrane Knowledge Translation framework. Regarding prioritization and co-production: Media coverage on climate change and health was tracked for 3 years (2019–2021) in 13 national and regional high-circulation English-language newspapers, as well as Australian Broadcasting Corporation (ABC) online news, and transcripts in the Factiva media database. Over the period, it was noted that there was an increase of 1.5 times more in the total number of newspaper articles on climate change and health. However, there was a variation depending on location, where some cities had more coverage than others, with 71% mentioning “*pandemic*” in the context of climate change and health. In the past 3 years, the number of articles on how to adapt to a changing climate to reduce the health consequences has remained low. Additionally, scientific publications were tracked on climate change and health using the Scopus database. The results showed a 79% increase in the number of publications in 2021, where 55% were original research articles and the remainder were literature reviews and editorial articles. The majority of the studies were on health impact assessment, with fewer studies on adaptation and mitigation focusing on mental health, Infectious diseases, and exposures. There has been an increase in grant applications on climate change and health. Five-year funding of $10 million to the climate change and health field is expected to boost research capacity and capability across the country in the coming years (34).

Sustainable knowledge translation process: Furthermore, government engagement and involvement in climate change and health decision-making was tracked. Government engagement on the topic was evident through the existence of bills that mentioned climate change and health, such as the National Framework for Adaptation and Mitigation. This bill requires government officials to consider the impact of climate change on health in their line of duty. However, climate change and health were not mentioned in the legislation. Hence, there was a need for the government to develop a National Strategy on climate change, health, and wellbeing.

Additionally, Government engagement on climate change and health issues was assessed by looking at the parliamentary websites to observe whether and how the government was responding to climate change as a health issue. It was reported that there was an increase in engagement with the topic of climate change and health in 2021 compared to 2020; however, the engagement was not explicit on climate health as a primary focus.

The fourth knowledge translation approach to climate change and health was identified in the study by Fogg-Rogers et al. (33), and this approach involved engaging with general citizens of all ages in activities that provided them an opportunity to participate in climate change and health decision-making processes. Six engagement methods were executed in each of the six cities, and this approach met three themes of the Cochrane Knowledge Translation framework. Prioritization and co-production; (i) Skylines Game: participants were encouraged to balance different policies by acting as the Mayor of the city. (ii) Application: an application was provided that enabled citizens to monitor their transport activities, emission generation, and pollution exposure using mobile GPS data. Prioritization and co-production: (iii) School competition, which was done by engaging young people below the age of 18 years in a competition to select interventions they preferred to reduce emissions from housing, transport, and the use of resources. Prioritization and co-production: (iv) My City Videos: adults were invited to make films about the changes in their city, their mobility, and the steps they take to minimize their exposure. Prioritization and co-production: (v) Citizen Delphi process; iterative surveys and workshops were employed to recruit citizens as local experts in their cities. Citizen Delphi surveys generated qualitative examples of lived experiences and potential policy ideas, which were then voted on in a subsequent quantitative survey, and then discussed in diverse geographic and demographic community qualitative workshops around each city. Exchange: (vi) Mutual Learning Workshop where citizens were brought together with informed stakeholders with backgrounds in air pollution, carbon emissions, and health and wellbeing, to discuss the challenges facing the city and then co-create policy interventions for cleaner, healthier futures. Exchange: (vii) Stakeholder Dialogue Workshop, where citizens and stakeholders were brought back together to review and discuss the Delphi outcomes, Mutual Learning Workshop, and ClairCity Skylines evidence, and co-create scenarios for low-carbon, clean air, and healthy futures. (viii) Facilitating Pull, where during a Policy Workshop, scenarios generated in the Stakeholder Dialogue Workshop were quantified and then returned to the local stakeholders to agree on a single Unified Policy Scenario.

Lastly, the fifth knowledge translation approach was based on using climate change and health assessment tools to inform decision-making; this approach only met one theme of the Cochrane Knowledge Translation framework. Prioritization and co-production: Delloly et al. (29) reported using a quantitative health impact assessment tool to inform decision-making. The tool was developed within a consortium of research partners that brought together researchers decision-makers, and public groups in the development and use of evidence. This tool allowed for rapid comparison of city policies in terms of their impact on environmental exposures, population health, and greenhouse gas emissions. The results from the tool were used to inform decision-making processes and help prioritize policy options in discussions between the research team and policymakers.

## Discussion

This scoping review is deemed the first to explore the relationship between climate change and health research for decision-making and practice. Five studies published between 2020 and 2024 met the inclusion criteria, each describing distinct knowledge translation approaches for advancing climate change and health research into action. These studies highlighted various climate health risk areas of intervention using different knowledge translation approaches. The topics ranged from environmental exposures to extreme weather events, incorporating climate change into policy and legislation, urban transformation to improve health and wellbeing, the impacts of climate change on physical and mental health, and air pollution. The reported health outcomes were primarily physical and psychological health risks, as well as increased susceptibility to infectious diseases. The articles, however, did not explore in-depth the impacts of climate change on mental health, physical health, or infectious disease but suggested the links between climate change and these health impacts.

Across the five studies, the level of stakeholder engagement varied. In some cases, policymakers were actively involved in co-design processes or were consulted to align research with strategic priorities. In other cases, citizens were engaged through participatory tools and dialogue-based formats. Several studies pointed out limitations such as resource constraints, limited institutional support, and legislative or cultural barriers affecting the use of evidence in decision-making.

Notably, all identified studies were conducted in high-income countries. Even though none of the studies were conducted in LMICs, there are still lessons that we can draw from these studies, firstly, in advocating for more studies to explore the links between climate change and health in LMICs. And further exploring how these studies can inform decision-making. This could contribute to developing knowledge translation strategies that promote adaptation in local communities and contribute to building climate-resilient health systems.

The connection between climate change and both mental health and climate change and infectious diseases is well-researched (35, 36) but similar reviews emphasize that most of these studies also come from high-income countries. As global attention on the health impacts of climate change grows, understanding how climate change research is translated into policy and practice is critical for safeguarding population health. Incorporating a climate change lens in health planning is essential to make health systems more resilient and better equipped to address climate change-related health risks. Moreover, existing legislation can serve as a foundation for involving decision-makers in climate change and health-related policy.

Cities, too, can be transformed to meet environmental imperatives while enhancing public health and wellbeing. For instance, China employs smart technology to monitor air pollution in real-time, providing citizens with the information needed to protect their health (37). In Seoul, South Korea, a framework uses data from sensors to improve thermal comfort in urban areas prone to heat waves (38). Similarly, in Freiburg, Germany, urban design incorporates techno-ecological solutions such as grass-covered tram corridors with pervious surfaces, part of a water-sensitive design that promotes public transport and stormwater management (39, 40).

Another key strategy reported in this manuscript is tracking media coverage on climate change and health, as well as monitoring scientific publications and national government engagement. The authors noted an increase in coverage on the topic over the years. Furthermore, Cortés and Quiroga (41) reported a similar trend of increased coverage on the topic in Chile. While communicating the connection between climate change and health is important, it should be noted that it is only the first step toward provoking change and eliciting responses from the public, politicians, and other key stakeholders (42). This strategy can be applied in LMICs, perhaps through a knowledge translation platform dedicated to tracking literature and engagement on climate change and health.

Citizen engagement was found useful in addressing local challenges by involving citizens in co-creating policy interventions. Engaging citizens in co-creating climate change and health policy is rarely reported in the literature; however, there are studies on citizen engagement in risk communication before, during, and after extreme weather events like flooding and drought (43, 44). A lesson from the included studies is that efforts to engage local communities in climate and health decision-making should involve activities tailored to different groups; these activities should also be enjoyable to improve their effectiveness (33).

Furthermore, the combination of knowledge generation, synthesis, and dissemination plays a vital role in informing decisions. This is more of a traditional form of knowledge translation, which could be complemented by other activities described in this manuscript. The field of knowledge translation is evolving, with increasing emphasis on integrated knowledge translation, a process that is dynamic, interactive, and nonlinear, moving beyond a reductionist view to attain inter-, multi-, and transdisciplinary collaboration (45).

The fourth strategy highlights the importance of advocacy and education in ensuring the optimal use of climate change research in health decision-making. Building collaborations and partnerships can secure buy-in and enable research to inform decision-making effectively. Other authors also speak about training health professionals and researchers in climate change communication and advocacy to ensure that the processes unfold effectively (46). Finally, conducting health impact assessments for informed decisions on climate change and health has been demonstrated in France as a valuable adaptation strategy. Moreover, the use of technology to facilitate decision-making aligns with the concept of smart cities, as defined by Nam and Pardo (47), which emphasizes the capacity of smart cities to collect and utilize data for effective decision-making.

## Conclusion

Our findings highlighted key knowledge translation approaches used in climate change and health decision-making and practice. These approaches reflect both stakeholder experiences and the engagement strategies employed to bridge the gap between research and action. There is a significant lack of information on knowledge translation, climate change, and health decision-making in LMICs, despite these countries hosting the most vulnerable communities affected by climate change. The issue of climate equity and inclusion is crucial, as LMICs, which contribute minimally to global warming, face greater impacts. These countries should be able to share in the economic and environmental benefits derived from climate action.

Based on the findings from this scoping review, we propose the following strategies for LMICs to promote the translation of climate change and health research into policy and action. We recommend establishing a knowledge translation platform that can monitor literature on climate change and health, including academic publications, newspapers, online articles, social media, websites, and other relevant sources that publish related information, to identify what exists and what is being published. Additionally, forming partnerships with stakeholders and utilizing advocacy and education can help obtain their buy-in and encourage their involvement in climate health adaptation strategies. Moreover, employing interactive and engaging activities when engaging with stakeholders, with different activities tailored for various groups or audiences, can improve participation. Finally, developing context-sensitive assessment tools to generate local data and using these tools to guide decision-making is crucial.

The study limitations include not having found any studies on knowledge translation and climate change in LMICs. Although we searched for global literature, the studies that met the inclusion criteria were all from high-income countries and mostly Europe-based. However, the findings from this review are still applicable to LMICs.

## Data Availability

The raw data supporting the conclusions of this article will be made available by the authors without undue reservation.

## Footnotes

^1^ https://www.covidence.org/

^2^ www.atlasti.com
