# Supplementary material for: Knowledge translation of climate change research into public health action: a scoping review
Source: Front Public Health. 2025 Oct 27;13:1632799. doi: 10.3389/fpubh.2025.1632799 (PMC12597920; doi:10.3389/fpubh.2025.1632799)
Supplement: Supplementary file 1 [file Table_1.DOCX]

Supplementary file 1

Box 1

PubMed search string

Search: (#5) NOT (#6)

(("climate change"[MeSH Terms] OR ("climate"[All Fields] AND "change"[All Fields]) OR "climate change"[All Fields] OR ("global warming"[MeSH Terms] OR ("global"[All Fields] AND "warming"[All Fields]) OR "global warming"[All Fields])) AND (("global"[All Fields] OR "globalism"[All Fields] OR "globalize"[All Fields] OR "globalized"[All Fields] OR "globalizes"[All Fields] OR "globalizing"[All Fields] OR "globally"[All Fields] OR "globals"[All Fields] OR "internationality"[MeSH Terms] OR "internationality"[All Fields] OR "globalization"[All Fields] OR ("high-income"[All Fields] AND ("countries"[All Fields] OR "country"[All Fields] OR "country s"[All Fields] OR "countrys"[All Fields])) OR ("developing countries"[MeSH Terms] OR ("developing"[All Fields] AND "countries"[All Fields]) OR "developing countries"[All Fields] OR ("low"[All Fields] AND "income"[All Fields] AND "country"[All Fields]) OR "low income country"[All Fields]) OR ("developing countries"[MeSH Terms] OR ("developing"[All Fields] AND "countries"[All Fields]) OR "developing countries"[All Fields] OR ("middle"[All Fields] AND "income"[All Fields] AND "country"[All Fields]) OR "middle income country"[All Fields]) OR "LMIC"[All Fields]) AND ("translational science, biomedical"[MeSH Terms] OR ("translational"[All Fields] AND "science"[All Fields] AND "biomedical"[All Fields]) OR "biomedical translational science"[All Fields] OR ("knowledge"[All Fields] AND "translation"[All Fields]) OR "knowledge translation"[All Fields] OR ("translational science, biomedical"[MeSH Terms] OR ("translational"[All Fields] AND "science"[All Fields] AND "biomedical"[All Fields]) OR "biomedical translational science"[All Fields] OR ("translational"[All Fields] AND "science"[All Fields]) OR "translational science"[All Fields]) OR (("knowledge"[MeSH Terms] OR "knowledge"[All Fields] OR "knowledge s"[All Fields] OR "knowledgeability"[All Fields] OR "knowledgeable"[All Fields] OR "knowledgeably"[All Fields] OR "knowledges"[All Fields]) AND ("uptake"[All Fields] OR "uptakes"[All Fields] OR "uptaking"[All Fields])) OR (("research personnel"[MeSH Terms] OR ("research"[All Fields] AND "personnel"[All Fields]) OR "research personnel"[All Fields] OR "researcher"[All Fields] OR "researchers"[All Fields] OR "research"[MeSH Terms] OR "research"[All Fields] OR "research s"[All Fields] OR "researchable"[All Fields] OR "researche"[All Fields] OR "researched"[All Fields] OR "researcher s"[All Fields] OR "researches"[All Fields] OR "researching"[All Fields] OR "researchs"[All Fields]) AND ("uptake"[All Fields] OR "uptakes"[All Fields] OR "uptaking"[All Fields])) OR (("evidence"[All Fields] OR "evidences"[All Fields] OR "evident"[All Fields] OR "evidently"[All Fields]) AND ("inform"[All Fields] OR "informal"[All Fields] OR "informality"[All Fields] OR "informally"[All Fields] OR "informant"[All Fields] OR "informant s"[All Fields] OR "informants"[All Fields] OR "information"[All Fields] OR "information s"[All Fields] OR "informational"[All Fields] OR "informations"[All Fields] OR "informative"[All Fields] OR "informatively"[All Fields] OR "informativeness"[All Fields] OR "informativity"[All Fields] OR "informed"[All Fields] OR "informer"[All Fields] OR "informers"[All Fields] OR "informing"[All Fields] OR "informs"[All Fields]) AND ("decision making"[MeSH Terms] OR ("decision"[All Fields] AND "making"[All Fields]) OR "decision making"[All Fields])) OR (("evidence"[All Fields] OR "evidences"[All Fields] OR "evident"[All Fields] OR "evidently"[All Fields]) AND ("inform"[All Fields] OR "informal"[All Fields] OR "informality"[All Fields] OR "informally"[All Fields] OR "informant"[All Fields] OR "informant s"[All Fields] OR "informants"[All Fields] OR "information"[All Fields] OR "information s"[All Fields] OR "informational"[All Fields] OR "informations"[All Fields] OR "informative"[All Fields] OR "informatively"[All Fields] OR "informativeness"[All Fields] OR "informativity"[All Fields] OR "informed"[All Fields] OR "informer"[All Fields] OR "informers"[All Fields] OR "informing"[All Fields] OR "informs"[All Fields])) OR ("sci commun"[Journal] OR "jcom j sci commun"[Journal] OR ("science"[All Fields] AND "communication"[All Fields]) OR "science communication"[All Fields])))) NOT ("biomedical"[All Fields] OR "biomedically"[All Fields] OR ("biomedical"[All Fields] OR "biomedically"[All Fields]) OR "biomed"[All Fields])
